# ERF72 interacts with ARF6 and BZR1 to regulate hypocotyl elongation in Arabidopsis

**DOI:** 10.1093/jxb/ery220

**Published:** 2018-06-12

**Authors:** Kun Liu, Yihao Li, Xuena Chen, Lijuan Li, Kai Liu, Heping Zhao, Yingdian Wang, Shengcheng Han

**Affiliations:** Beijing Key Laboratory of Gene Resource and Molecular Development, College of Life Sciences, Beijing Normal University, Beijing, China

**Keywords:** ERF72, ARF6, BZR1, hypocotyl elongation, photomorphogenesis, *Arabidopsis thaliana*

## Abstract

The phytohormones brassinosteroid (BR), auxin, and gibberellin (GA) regulate photomorphogenesis-related hypocotyl elongation in Arabidopsis via the co-operative interaction of BZR-ARF-PIF/DELLA (BAP/D) transcription factors/regulators. In addition, ethylene activates the PIF3 or ERF1 pathway through EIN3/EIL1 to balance hypocotyl elongation in Arabidopsis seedlings. However, the mechanism by which ethylene is co-ordinated with other phytohormones to produce light-regulated hypocotyl growth remains elusive. In this study, we found that hypocotyl cell elongation is regulated by a network involving ethylene, auxin, and BR signalling, which is mediated by interactions among ERF72, ARF6, and BZR1. ERF72 interacted directly with ARF6 and BZR1 *in vitro* and *in vivo*, and it antagonised regulation by ARF6 and BZR1 of the transcription of *BEE3* and *XTH7*. In addition, light modulated the subcellular localisation of ERF72 and transcription of *ERF72* through the EIN2-EIN3/EIL1 pathway, facilitating the function of ERF72 in photomorphogenesis. The expression of *BEE3* and *XTH7* was also regulated by the EIN2-EIN3/EIL1 pathway. Our findings indicate that a revised BZR-ARF-PIF/DELLA-ERF (BAP/DE) module integrates light and hormone signals to regulate hypocotyl elongation in Arabidopsis.

## Teaser

Hypocotyl cell elongation related to photomorphogenesis in Arabidopsis seedlings is regulated by a network involving ethylene, auxin, and brassinosteroid signalling that is mediated by interactions among ERF72, ARF6, and BZR1, forming a revised BZR-ARF-PIF/DELLA-ERF (BAP/DE) module.

## Introduction

After germination, plants exhibit two distinct adaptations to the surrounding environment. Seedlings grown in darkness exhibit elongated hypocotyls and the formation of an apical hook, while development of the cotyledons, apical meristem, and root system is inhibited; this is termed skotomorphogenesis. In contrast, seedlings grown in light and undergoing photomorphogenesis are characterised by inhibited hypocotyl elongation, accelerated root growth, opened and green cotyledons, and no apical hook (Von Arnim and Deng, 1996). Both skotomorphogenesis and photomorphogenesis are driven by cell division in the apical meristems followed by cell elongation, resulting in growth of the hypocotyl and the roots (Chaiwanon *et al.*, 2016; de Wit *et al.*, 2016). Light signals are perceived by different photoreceptors and are transmitted to downstream transcription factors in order to regulate cell elongation during seedling morphogenesis. There are two classes of primary photoreceptor-regulated transcription factors, which play opposite roles in regulating cell elongation. The transcription factors ELONGATED HYPOCOTYL5 (HY5) and GATA2/4, which are degraded by the E3 ubiquitin ligase CONSTITUTIVE PHOTOMORPHOGENIC1 (COP1) under dark conditions, negatively regulate cell elongation (Luo *et al.*, 2010; Huang *et al.*, 2014). Many targets of HY5 are regulators of hormone signalling, including those of abscisic acid (ABA), gibberellin (GA), ethylene, auxin, brassinosteroid (BR), cytokinin, and jasmonic acid (Lau and Deng, 2010; Wang *et al.*, 2012; Oh *et al.*, 2014). Another class of transcription factors, PHYTOCHROME INTERACTION FACTORS (PIFs), positively regulate cell elongation in darkness and are rapidly degraded upon exposure to light (Leivar *et al.*, 2008; Leivar and Monte, 2014). Previous studies have shown that PIFs integrate light signals, hormone-signalling pathways, and the circadian clock to optimise cell elongation and seedling photomorphogenesis (Leivar and Monte, 2014; Paik *et al.*, 2017).

The signalling pathways centred on the plant growth-promoting hormones auxin, BR, GA, and ethylene have been studied extensively (Kim and Wang, 2010; Sun, 2010; Merchante *et al.*, 2013; Weijers and Wagner, 2016). Auxin is perceived by a co-receptor comprising a TRANSPORT INHIBITOR-RESISTANT 1/AUXIN SIGNALLING F-BOX (TIR1/AFB) protein and an AUXIN/INDOLE-3-ACETIC ACID (Aux/IAA) transcriptional co-regulator (Calderón Villalobos *et al.*, 2012), leading to polyubiquitination and degradation of the Aux/IAA protein and the release of AUXIN RESPONSE FACTOR (ARF) family transcription factors to activate auxin-responsive transcription (Weijers and Wagner, 2016). SHORT HYPOCOTYL2 (SHY2)/IAA3 is a member of the Aux/IAA family in Arabidopsis, and 1-week-old seedlings of *shy2-2* grown in the dark show short hypocotyls, expanded cotyledons, open apical hooks, and true leaf primordia, suggesting an important role for auxin in regulating photomorphogenesis (Tian and Reed, 1999). In addition, a double-mutant of *ARF6* and its close homolog *ARF8*, *arf6 arf8*, shows a short-hypocotyl phenotype in darkness, suggesting that ARF6 and ARF8 regulate hypocotyl elongation redundantly (Nagpal *et al.*, 2005).

BR binds the receptor kinase BRASSINOSTEROID-INSENSITIVE1 (BRI1) at the cell surface and transduces the signal to activate the transcription factors BRASSINOZALE-RESISTANT1 (BZR1) and BZR2 (also named BES1 for BRI1-EMS-SUPPRESSOR1), which regulate the expression of BR-responsive genes (He *et al.*, 2005; Kim and Wang, 2010). Arabidopsis BR-insensitive or -deficient mutants display a de-etiolated phenotype under dark conditions (Clouse *et al.*, 1996; Li *et al.*, 1996; Clouse, 2002), and the light-induced genes were de-repressed in BR-deficient mutants under dark conditions (Szekeres *et al.*, 1996; Song *et al.*, 2009). In addition, the constitutive photomorphogenesis phenotype of BR-deficient or -insensitive mutants is suppressed by the dominant *bzr1-1D* and *bes1-D* mutations, indicating that activation of BZR1 and BZR2 is essential for skotomorphogenesis in Arabidopsis (Wang *et al.*, 2002; Yin *et al.*, 2002).

GA, similar to auxin, regulates cell elongation by de-repressing its signalling pathway via intracellular receptor GIBBERELLIN-INSENSITIVE DWARF1 (GID1)-induced degradation of DELLA proteins via the ubiquitin-proteasome pathway (Sun, 2010). DELLAs were first found to interact with PIFs and inhibit their DNA-binding activity (de Lucas *et al.*, 2008; Feng *et al.*, 2008), and subsequently reported to inhibit the DNA-binding activities of many transcription factors, including BZR1 and ARF6 (Bai *et al.*, 2012; Oh *et al.*, 2014). The interactions of ARF6, BZR1, and PIF4 enhance their target-binding and transcriptional activities, and these factors synergistically promote hypocotyl elongation by co-activating numerous shared target genes with known functions in cell elongation (*EXP8*, *BIM1*, *BEE1/3*, *PREs*, *HAT2*, *IBH1*, *HFR1*, *PAR1/2*, and *EXO*) (Oh *et al.*, 2014). The interactions among ARF6, BZR1, and PIF4, and the inhibition of their function by DELLAs, is known as the BZR-ARF-PIF/DELLA (BAP/D) transcriptional module, and it elegantly mediates the co-operative regulation by BR, auxin, GA, and light signals of cell elongation during seedling morphogenesis (Bai *et al.*, 2012; Oh *et al.*, 2014; Chaiwanon *et al.*, 2016).

Ethylene is perceived by five endoplasmic reticulum-localised receptors, ETHYLENE RECEPTOR1 (ETR1), ETR2, ETHYLENE RESPONSE SENSOR1 (ERS1), ERS2, and ETHYLENE-INSENSITIVE4 (EIN4), that relieve repression by CONSTITUTIVE TRIPLE RESPONSE1 (CTR1) of the downstream signalling component EIN2 (Gallie, 2015). EIN2 is cleaved and translocates to the nucleus, where it stabilises EIN3 and its homolog EIN3-LIKE1 (EIL1) and thus activates the transcription of ethylene-responsive genes in Arabidopsis (Ju *et al.*, 2012; Qiao *et al.*, 2012; Wen *et al.*, 2012). Ethylene activates two pathways through EIN3/EIL1 to synchronously regulate hypocotyl growth and cotyledon development in response to different light and soil conditions in Arabidopsis seedlings (Zhong *et al.*, 2012). One of these pathways depends on the induction of *ETHYLENE RESPONSE FACTOR1* (*ERF1*), and the other depends on the induction of *PIF3*; both are direct targets of EIN3. Under dark conditions, PIFs promote hypocotyl elongation, and soil-dependent ethylene accumulation leads to ERF1-mediated thickening of the hypocotyl cell wall and inhibition of etiolated hypocotyl elongation. The increased level of ethylene also represses biosynthesis of the chlorophyll precursor protochlorophyllide in the cotyledons via the PIF3 pathway. Protochlorophyllide, when present at high levels, can cause photo-oxidative damage after light absorption. However, when seeds germinate in the presence of light or when seedlings emerge from the soil, PIFs are degraded and the ethylene-induced increased abundance of PIF3 stimulates hypocotyl elongation. In contrast, ERF1 is stabilised in the presence of light, and further stimulation of ERF1 by ethylene has no effect on growth (Zhong *et al.*, 2012). These results explain the ethylene-induced response in dark-grown seedlings, and suggest that ethylene plays an important role in co-ordinating growth and development to guide emergence from the soil and produce the shift to photoautotrophic growth (Ecker, 1995; Benavente and Alonso, 2006; de Wit *et al.*, 2016). However, the links between ethylene and other hormones that regulate photomorphogenesis are unclear.

In this study, we found that transgenic Arabidopsis seedlings overexpressing stable *ERF72* (*35S::MAERF*) exhibited the constitutive photomorphogenesis phenotype of a short hypocotyl, no apical hook, and open cotyledons in darkness, compared with the wild-type, the *erf* mutant, and plants overexpressing wild-type *ERF72* (*35S::MCERF*). Transcriptomic analyses showed that light-regulated genes and ARF6- and BZR1-target genes were enriched among ERF72-related differentially expressed genes. We further demonstrated that ERF72 interacted with ARF6 and BZR1 to repress their activation of the transcription of target genes. Moreover, light modulated the subcellular localisation of ERF72 and the transcription of *ERF72* through the EIN2-EIN3/EIL1 pathway. The expression of *BEE3* and *XTH7* was also regulated by the EIN2-EIN3/EIL1 pathway under any light regimen. Our results suggest that a revised BZR-ARF-PIF/DELLA-ERF (BAP/DE) module is involved in integrating light and hormone signalling pathways to control cell elongation in Arabidopsis hypocotyls.

## Materials and methods

### Plant growth and phenotypic analyses

The *Arabidopsis thaliana* plants used in this study were of the Col-0 ecotype. The T-DNA insertion mutant *erf* (Stock No: CS849696) was obtained from The Arabidopsis Information Resource (TAIR; https://www.arabidopsis.org/) and confirmed by genotyping, PCR, and RT-PCR (Supplementary Fig. S1 at *JXB* online). All seeds were surface-sterilised and sown on half-strength Murashige and Skoog (MS) medium containing 1.5% sucrose and 0.7% agar, and incubated at 4 °C under dark conditions for 3 d. The plates were then irradiated with white light for 6 h to promote germination and subsequently kept either in darkness set periods of time or under continuous red light (60 µmol m^–2^ s^–1^), far-red light (5 µmol m^–2^ s^–1^), blue light (7 µmol m^–2^ s^–1^), or white light (60 µmol m^–2^ s^–1^) for 5 d. For the light-to-dark transition experiments, seedlings were grown under continuous light or dark conditions for 7 d, and then transferred to the opposite conditions for set periods of time. For cycloheximide (CHX) treatment, seedlings were grown in continuous light for 7 d, transferred to plates containing half-strength MS medium with or without 100 μM CHX, and then placed in the light or darkness for set periods of time. The seedlings were then collected for experimental use.

Images of the hook and hypocotyl phenotypes of seedlings were obtained using an EOS60D digital camera (Canon, Japan). Cell length was determined using a confocal laser-scanning microscope (LSM700; Zeiss, Germany). Hypocotyl length and cell length were measured using the ImageJ software (https://imagej.nih.gov/ij/).

### Plasmid construction

The full-length cDNAs of the wild-type (WT) ERF72 (*MCERF*) and stable *ERF72* (with the second amino acid, cysteine, of ERF72 mutated to alanine; *MAERF*) lacking a stop codon were amplified by RT-PCR and inserted into the *Bam*HI and *Not*I restriction sites of the plasmid *pE2c* (Addgene; http://www.addgene.org/), which harbours a triple HA-tag, to generate the construct *pE2c-MC(/A)ERF72-HA*. The *MAERF72* fragment was inserted into the same restriction sites of *pE6c* (Addgene) containing a yellow fluorescent protein (YFP) tag to generate the construct *pE6c-MAERF-YFP*. In addition, the *MC(/A)ERF72* fragment was inserted into *pMDC32* using the Gateway LR II kit (Invitrogen, USA) to generate the *35S::MC(/A)ERF-HA* and *35S::MAERF-YFP* constructs. To generate a native promoter-driven plant expression vector, the 1443-bp DNA fragment upstream of the ATG codon of *ERF72* was amplified and used to replace the 35S promoter of the *35S::MAERF* construct to generate *P*_*ERF72*_*::MAERF*. The full-length cDNAs of *ARF6* and *BZR1* lacking a stop codon were amplified by RT-PCR and inserted into the *Bam*HI and *Not*I restriction sites of *pE3c* (Addgene), which harbours a triple MYC-tag, to generate the constructs *pE3c-ARF6* and *pE3c-BZR1*, respectively.

For promoter activity assays, the 2553-bp DNA fragment upstream of the ATG codon of *BEE3* was amplified and inserted into the *Bam*HI and *Asc*I restriction sites of *pGPTV* to generate the *P*_*BEE3*_*::GUS* (β-glucuronidase) construct. In addition, the 2023-bp DNA fragment upstream of the ATG codon of *XTH7* was amplified and inserted into the *Hin*dIII and *Asc*I restriction sites of *pGPTV* to generate the *P*_*XTH7*_*::GUS* construct. The promoter fragment of *ERF72* was cut from *P*_*ERF72*_*::MAERF* and inserted into the *Hin*dIII and *Asc*I restriction sites of *pGPTV* to generate the *P*_*ERF72*_*::GUS* construct. For the 4×GCC-like box construct, DNA fragments containing the G1 or G2 box from the *BEE3* promoter and G3 or G4 box from the *XTH7* promoter were synthesised and inserted into the *Asc*I and *Xba*I sites or the *Sal*I and *Xba*I sites of *pE1n*, respectively. Using two pairs of isocaudomers, *Spe*I and *Xba*I in pE1-G1/G2 or *Sal*I and *Xho*I in pE1-G3/G4, four copies of the GCC-like box fragments were made and inserted into the *Asc*I and *Xba*I restriction sites of *pGPTV* to generate the *P*_4×G1_*::GUS* or *P*_4×G2_*::GUS* constructs, or into the *Hin*dIII and *Sal*I restriction sites of *pGPTV* to generate the *P*_4×G3_*::GUS* or *P*_4×G4_*::GUS* constructs.

To produce constructs for yeast two-hybrid assays, truncated fragments of *ARF6*, *ARF6N*, and *ARF6C* were subcloned from *pE3c-ARF6* and inserted into the *Bam*HI and *Not*I restriction sites of *pE1c* to generate the *pE1c-ARF6N* and *pE1c-ARF6C* constructs. The *ARF6N/ARF6C* fragment was inserted into *pDEST-GBKT7* using the Gateway LR II kit to generate the *pGBKT7-ARF6N/ARF6C* construct. The full-length cDNA of *ERF72* was amplified by PCR and inserted into *pGADT7* using *Eco*RI and *Bam*HI restriction sites to generate *pGADT7-ERF72*.

To construct vectors for the production of recombinant proteins, the *BZR1-MYC* fragment of *pE3c-BZR1* was inserted into *p28a-DEST* using the Gateway LR II kit to generate the p*ET28a-BZR1-MYC* construct, and the *ERF72* fragment from *pE2c-MCERF72-HA* was inserted into *pHIS-6p-MBP* using the *BamHI* and *NotI* restriction sites to generate *pHIS-MBP-ERF*.

To construct vectors for firefly luciferase (LUC) complementation imaging (LCI) assays (Chen *et al.*, 2008), *ARF6* and *BZR1* fragments were digested from *pE3c-ARF6/BZR1* by *Bam*HI and *Xho*I or *Bam*HI and *Xba*I, and subcloned into *35S::NLuc* digested with *Bam*HI and *Xho*I or *Bam*HI and *Xba*I to generate *35S::ARF6/BZR1-NLuc*. The *ERF72* fragment was inserted into the *Kpn*I and *Sal*I restriction sites of *35S::CLuc* to generate the *35S::CLuc-ERF* vector.

All clones and vectors were validated by sequencing. The primers used are listed in Supplementary Table S1.

### Generation of transgenic plants

The constructs *35S::MC(/A)ERF*, *35S::MAERF-YFP*, and *P*_*ERF72*_*::MAERF* were separately introduced into *Agrobacterium* GV3101, and then transformed into WT Arabidopsis plants to generate the *35S::MCERF*, *35S::MAERF*, and *35S::MAERF-YFP* transgenic lines, or into the *erf* mutant to generate the *P*_*ERF72*_*::MAERF* transgenic line, using the floral dip transformation method (Clough and Bent, 1998). The transformants were selected on half-strength MS medium containing 50 mg ml^–1^ hygromycin B (Sigma-Aldrich, USA). T3-generation homozygous transformants carrying a single insertion were used in subsequent experiments.

### RNA-seq analysis

Total RNA was extracted from seedlings grown under dark conditions at 2 d after gemination (DAG) using an RNA Extraction Kit (Promega, USA). A total of 3.0 µg of RNA was used to generate sequencing libraries using the NEBNext Ultra RNA Library Prep Kit following the manufacturer’s instructions (NEB, USA), and index codes were added to attribute sequences to each sample. Solexa sequencing was performed as a commercial service at Novogene (http://www.novogene.com/) with an Illumina HiSeq 2000 sequencer. The sequencing data were deposited in the NCBI’s Sequence Read Archive (SRA; https://www.ncbi.nlm.nih.gov/sra) under accession number SRP125848. Low-quality bases (*Q*<20) at the ends of the sequencing reads were trimmed using the SolexaQA software (Cox *et al.*, 2010) (ver. 1.10, parameters: –b h 20); our RNA-seq data are shown in Supplementary Table S2. After trimming, all reads were mapped to the Arabidopsis genome (TAIR9; www.arabidopsis.org) using the TopHat software (Trapnell *et al.*, 2009). Read counts were generated using HTSeq in union mode. Differentially expressed genes (DEGs) between samples were defined by Deseq (Anders and Huber, 2010), using a fold-change >2 and adjusted *P*-value <0.05.

### Gene expression analysis

For qRT-PCR analysis, total RNA was isolated using an RNA Extraction Kit (Promega, USA), and first-strand cDNA was synthesised from 3.0 µg of RNA using a reverse transcriptase (TransGen, China). qRT-PCR was performed using TransStart Tip Green qPCR Super Mix following the manufacturer’s instructions (TransGen). Three biological replicates were performed per sample, and expression levels were normalised to that of *Actin2* as the control. Value changes of more than two-fold (>2 or <0.5) were considered to indicate a significant difference of target gene expression. The primers used are listed in Supplementary Table S1.

### Yeast two-hybrid assay

The GAL4 DNA-binding domain (BD) fusion plasmid *pGADT7-ERF72* and the activation domain (AD) fusion plasmid *pGBKT7-ARF6N/ARF6C* were transformed into *Saccharomyces cerevisiae* AH109. Different combinations of AD and BD fusion plasmids were generated by mating. Yeast strains were grown on SD/–Trp–Ura–His dropout plates containing 1 mM 3-amino-1,2,4-triazole (3-AT) to confirm their interactions.

### Recombinant protein production and pull-down assays

BZR1-MYC and MBP-ERF fusion proteins were expressed in *Escherichia coli* BL21 (DE3) and induced by isopropyl β-D-1-thiogalactopyranoside (IPTG). Briefly, *E. coli* BL21 cells containing p*ET28a-BZR1-MYC* and *pHIS-MBP-ERF* were induced for 8 h with 0.1 mM IPTG at 16 °C, and subjected to ultrasonic treatment in lysis buffer (50 mM Tris-HCl pH 7.4, 200 mM NaCl, 1 mM β-mercaptoethanol, 0.5% Triton X-100, and 10% glycerine). After centrifugation, the supernatant was used as the crude protein extract.

For pull-down assays, crude protein extracts were added to binding buffer (10 mM Tris-HCl pH 7.5, 150 mM NaCl, 2 mM KCl, and 0.1% Tween-20), followed by incubation at 4 °C for 1 h with gentle rotation. Amylose resin (20 μl) was added, followed by incubation for 1 h to precipitate MBP-containing proteins. The resin was washed five times in binding buffer.

### Immunoblot assays

Seedlings were ground to powder in liquid nitrogen, and total proteins were extracted in buffer [62.5 mM Tris-HCl pH 6.8, 2% sodium dodecyl sulphate (SDS), 10% glycerine, and 1 mM β-mercaptoethanol]. The protein concentration was determined using a Bio-Rad Protein Assay Kit with bovine serum albumin as the standard. Following resolution by SDS-polyacrylamide gel electrophoresis, proteins were transferred to a polyvinylidene difluoride membrane (Millipore, USA) under 200 mA constant current for 45 min in transfer buffer (12.5 mM Tris-HCl, 192 mM glycine, and 10% methanol; pH 8.3). Anti-HA (Abcam, 1:10 000) and anti-MYC (Cell Signalling Technology, 1:1000) antibodies were used to visualise protein bands.

### LCI assays

LCI assays were carried out as described previously (Chen *et al.*, 2008). *Agrobacterium* cells containing constructs were suspended in infiltration medium (MS medium containing 10 mM MES pH 5.6, and 150 mM acetosyringone) to an optical density at 600 nm (OD_600_) of 1.0, and every pair of constructs were mixed in equimolar ratios. Luciferase activity was assayed using a Lumazone FA1300 Imaging System (Roper Scientific, USA).

### ChIP-qPCR assays

Chromatin immunoprecipitation (ChIP)-qPCR assays were performed as described previously (Cui *et al.*, 2016). Briefly, 5 ml of protoplasts were transfected with ARF6-MYC or BZR1-MYC alone or together with ERF72-HA, and incubated for 24 h. Cell walls were cross-linked in 1% formaldehyde for 20 min and quenched in glycine for 5 min. Chromatin complexes were isolated and sonicated to reduce the average DNA fragment size to ~500 bp. An anti-MYC antibody (1:50) was used to pull down DNA-protein complexes. The precipitated DNA fragments were recovered and quantified by qPCR using SYBR Premix ExTaq Mix. The relative enrichment of DNA was calculated by normalising the amount of target DNA to that of the internal control gene *CNX5* (At5g55130), and the input DNA amount. Data are presented as means (SD) of three biological replicates. The primers used for qPCR are listed in Supplementary Table S1.

### Transient gene expression assays

Arabidopsis mesophyll protoplasts (2 × 10^5^) were isolated from 4-week-old rosette leaves and transfected with 30 μg of DNA (effector:reporter:internal standard = 5:4:1) and incubated overnight as described previously (Liang *et al.*, 2015). Protoplasts were harvested by centrifugation and lysed in 100 μl of passive lysis buffer (Promega, USA). Firefly luciferase activity (as an internal standard) was measured using a Luciferase Reporter Kit (Promega, USA). GUS activity was measured as described previously (Jefferson *et al.*, 1987).

### Statistical analysis

Statistical analyses were performed using the Data Processing System (Tang and Zhang, 2013). One-way ANOVA and Tukey’s multiple range test were conducted to determine the significance of differences (*P*<0.05).

### Accession numbers

The RNA-seq data are deposited in the Sequence Read Archive at NCBI (https://www.ncbi.nlm.nih.gov/sra) under accession number SRP125848. Sequence data can be found in TAIR (https://www.arabidopsis.org/) under the following accession numbers: *ERF72* (At3g16770), *ARF6* (At1g30330), *BZR1* (At1g75080), *BEE3* (At1g73830), *XTH7* (At4g37800), *CNX5* (At5g55130), *UBC30* (At5g56150), *PP2A* (At1g69960), *EIN3* (At3g20770), and *Actin2* (At3g18780).

## Results

### Overexpression of stable *ERF72* in Arabidopsis triggers a constitutive photomorphogenic-like response under dark conditions

**Fig. 1.**
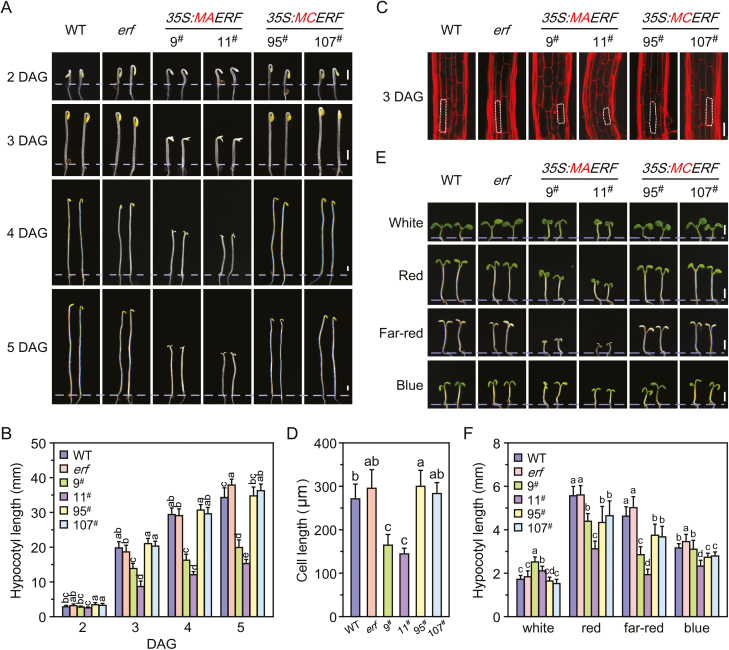
Overexpression of stabilised *ERF72* (*35S::MAERF*) in Arabidopsis triggers a constitutive photomorphogenic-like response. (A) Growth of wild-type (WT), *erf*, and transgenic seedlings under dark conditions. 9^#^ and 11^#^, independent transgenic lines overexpressing stable *ERF72* driven by the 35S promoter in a WT background (*35S::MAERF*); 95^#^ and 107^#^, independent lines overexpressing *ERF72* in a WT background (*35S::MCERF*). DAG, days after germination. Scale bars are 1 mm. (B) Hypocotyl length of WT, *erf*, *35S::MAERF*, and *35S::MCERF* seedlings under the same conditions as in (A). Data are means (±SD) of at least 20 seedlings. Different letters indicate significant differences at *P*<0.05 according to Tukey’s test. (C) Propidium iodide staining of hypocotyl cells of 3-DAG seedlings of WT, *erf*, *35S::MAERF*, and *35S::MCERF* under the same conditions as in (A). The scale bar is 100 μm. (D) Cell length of 3-DAG seedlings under the same conditions as in (C). Data are means (±SD) of at least 20 seedlings. Different letters indicate significant differences at *P*<0.05 according to Tukey’s test. (E) Growth of 5-DAG seedlings of WT, *erf*, *35S::MAERF*, and *35S::MCERF* under continuous white, red, far-red, and blue light. Transgenic lines are as in (A). Scale bars are 2 mm. (F) Hypocotyl length of WT, *erf*, *35S::MAERF*, and *35S::MCERF* seedlings under the light conditions described in (E). Data are means ( SD) of at least 20 seedlings. Different letters indicate significant differences at *P*<0.05 according to Tukey’s test.

In Arabidopsis, there are five Group-VII ERFs, namely HYPOXIA RESPONSIVE ERF1 (HRE1, ERF73), HRE2 (ERF71), RELATED TO AP2.12 (RAP2.12, ERF74), RAP2.2 (ERF75), and RAP2.3 (ERF72) (Nakano *et al.*, 2006), which are characterised by a conserved N-terminal domain beginning with the residues Met-Cys (MC) and which are involved in oxygen sensing and nitric oxide (NO)-regulated processes, including seed germination, stomatal closure, and hypocotyl elongation, via the N-end rule pathway of targeted proteolysis (Licausi *et al.*, 2011; Gibbs *et al.*, 2014). Here, we generated transgenic plants overexpressing WT *ERF72* (*35S::MCERF*) and proteolysis-resistant *ERF72* (with the second amino acid, cysteine, mutated to alanine; *35S::MAERF*) (Supplementary Fig. S1), and found that *35S::MAERF* plants exhibited a shorter hypocotyl, no apical hook, and more open cotyledons compared with WT, *erf* mutant, and *35S::MCERF* plants after germination under dark conditions (Fig. 1A, B, Supplementary Fig. S2). We determined the hypocotyl cell length of seedlings at 3 DAG and found that the mean length in *35S::MAERF* transgenic plants was significantly decreased compared with WT, *erf*, and *35S::MCERF* plants, which was in agreement with their shorter hypocotyls (Fig. 1C, D). These results demonstrated that the overexpression of stabilised *ERF72* in WT plants triggered a constitutive photomorphogenic-like response under dark conditions, and the resulting limited cell elongation contributed to the shorter hypocotyls of *35S::MAERF* plants.

We further evaluated seedling growth at 3 DAG under continuous red, far-red, blue, and white light conditions. The hypocotyl length of WT, *erf*, *35S::MCERF*, and *35S::MAERF* plants was significantly shorter under continuous red, far-red, and blue light conditions than under dark conditions, and the hypocotyl lengths of plants grown under white light were shortest compared with those grown under monochromatic light or dark conditions (Fig. 1E, F). More interestingly, the hypocotyls of *35S::MAERF* plants were slightly longer than those of WT, *erf*, and *35S::MCERF* plants grown under white light. However, the hypocotyls of *35S::MAERF*, similar to that of *35S::MCERF* plants, were shorter than those of WT and *erf* plants under continuous red and far-red light conditions (Fig. 1E, F), suggesting that the hypocotyl growth of *35S::MAERF* plants was dependent on the specific light conditions.

### RNA-seq analyses to identify ERF72-related DEGs

**Fig. 2.**
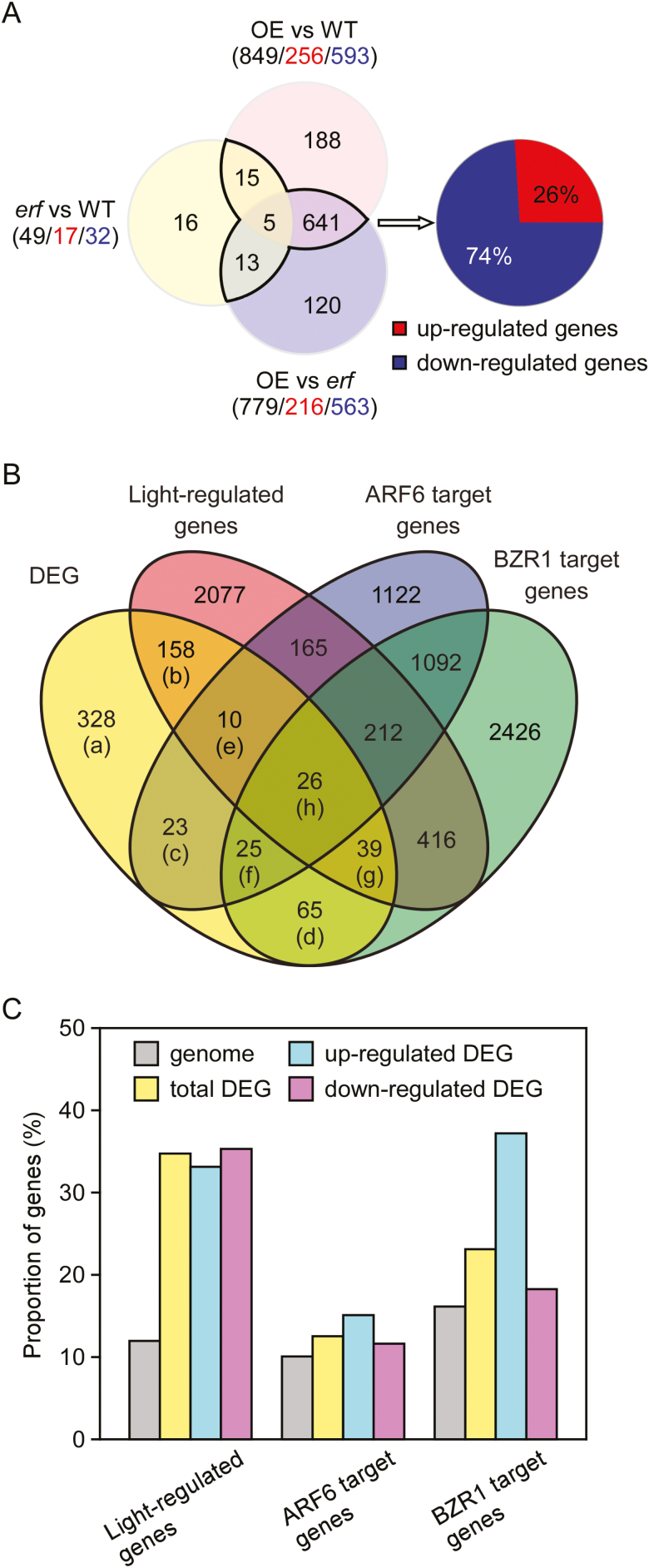
Light-regulated genes, ARF6-target genes, and BZR1-target genes are enriched in ERF72-responsive differentially expressed genes (DEGs). (A) Venn diagram of DEGs in dark-grown *35S::MAERF* plants (overexpressing, OE) versus wild-type (WT) (OE vs WT), OE vs *erf*, and *erf* vs WT based on the RNA-seq data. Black numbers, total numbers of DEGs; red and blue numbers, up-regulated and down-regulated genes, respectively. Genes within the black border are ERF72-responsive DEGs. (B) Venn diagram of ERF72-responsive DEGs, light-regulated genes, ARF6-target genes, and BZR1-target genes. (C) Enrichment of light-regulated genes, ARF6-target genes, and BZR1-target genes in ERF72-responsive DEGs. Genome indicates all genes.

To evaluate the role of ERF72 in controlling hypocotyl growth, we performed a transcriptomic analysis among WT, *erf*, and *35S::MAERF* plants grown under dark conditions. In total, 21 801 genes in the Arabidopsis genome were functionally annotated according to at least one read in any plant sample. Based on the fragments per kilobase of transcript per million mapped reads (FPKM) value of each gene using a cut-off of a two-fold change and an adjusted *P*-value <0.05 between two samples, 849 DEGs were identified between *35S::MAERF* (overexpressing, OE) and WT plants (256 up-regulated and 593 down-regulated), 779 DEGs between OE and *erf* plants (216 up-regulated and 563 down-regulated), and 49 DEGs between *erf* and WT plants (17 up-regulated and 32 down-regulated). Using a Venn diagram to analyse the DEGs of OE versus WT, OE versus *erf*, and *erf* versus WT plants based on the RNA-seq data, we identified 674 *ERF72*-related DEGs, including 176 up-regulated (26%) and 498 down-regulated genes (74%) (Fig. 2A, Supplementary Table S3).

### Light-regulated, and ARF6- and BZR1-target genes are enriched in ERF72-related DEGs

Previous studies have shown that ARF6 and its close homolog ARF8 regulate hypocotyl elongation (Nagpal *et al.*, 2005), and BZR1 promotes cell elongation and seedling morphogenesis in response to BR in Arabidopsis (Oh *et al.*, 2012). Therefore, the constitutive photomorphogenic-like phenotype of *35S::MAERF* seedlings led us to investigate the relationships between ERF72-related DEGs, light-regulated genes, ARF6-target genes, and BZR1-target genes. In our RNA-seq dataset, we found 2813 light-regulated genes, 2376 ARF6-target genes, and 3803 BZR1-target genes, among which 233 light-regulated genes, 84 ARF6-target genes, and 155 BZR1-target genes were ERF72-responsive DEGs (Fig. 2B, Supplementary Table S3). Furthermore, these three co-regulated gene categories were enriched dramatically among ERF-related DEGs compared to their representation in our RNA-seq dataset (Fig. 2C). More interestingly, the common target genes included several with known functions in cell elongation, such as *BEE1/3*, *PAR1/2*, and *EXO*, which were down-regulated in *35S::MAERF* seedlings (Supplementary Table S3). These results suggested that ERF72, ARF6, and BZR1 functioned co-operatively to regulate hypocotyl elongation and seedling photomorphogenesis.

### ERF72 physically interacts with ARF6 and BZR1

**Fig. 3.**
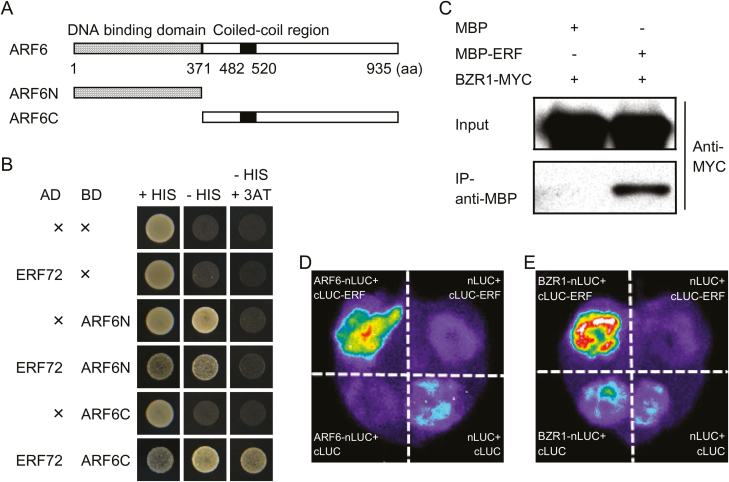
ERF72 interacts with ARF6 and BZR1. (A) Domain structures of ARF6 and its truncated fragments (ARF6N and ARF6C). (B) Yeast two-hybrid assay of activation domain (AD)-tagged ERF72 with binding domain (BD)-tagged ARF6N, and ARF6C or BD alone. (C) Pull-down assays of BZR1 and ERF72. Recombinant BZR1-MYC was used as prey and pulled down using protein crude extract of MBP or MBP-ERF72 as bait. (D, E) Firefly luciferase complementation imaging assays of the interaction of ERF72 with ARF6 (D) or BZR1 (E) in tobacco leaves. Full-length *ERF72* was fused to the C-terminal fragment of luciferase (c-LUC), and the full-length sequence of *ARF6* or *BZR1* was fused to the N-terminus of luciferase (n-LUC). Empty vectors were used as negative controls.

The significant enrichment of ARF6- and BZR1-target genes in ERF72-responsive DEGs suggested that ERF72 regulates their expression by activating or inhibiting the transcription of *ARF6* and *BZR1*, or by directly interacting with ARF6 and BZR1. We found that the expression levels of *ARF6* and *BZR1* in *35S::MAERF* plants were similar to those in WT and *erf* plants (fold-change <2) (Supplementary Fig. S3A), indicating that ERF72 had no effect on the transcription of *ARF6* and *BZR1*. Yeast two-hybrid assays using truncated fragments of ARF6 or BZR1 showed that ERF72 interacted with the C-terminal domain of ARF6 (Fig. 3A, B), while truncated fragments of BZR1 were capable of self-activation and did not interact with ERF72 (data not shown). Furthermore, a pull-down assay showed that MPB-tagged ERF72, but not MBP alone, pulled down recombinant MYC-tagged BZR1 *in vitro* (Fig. 3C), which demonstrated that ERF72 interacted with BZR1 *in vitro*. To confirm the interaction of ERF72 with ARF6 or BZR1 *in vivo*, a firefly LCI assay was performed in tobacco leaves. Co-expression of ARF6-nLUC fusion proteins with ERF72-cLUC or BZR1-nLUC with ERF72-cLUC yielded a strong fluorescence signal (Fig. 3D, E), suggesting that the functional luciferase was reconstituted via direct interactions between ERF72 and ARF6 or ERF72 and BZR1. These results suggested that ERF72 interacted with ARF6 or BZR1 to regulate the expression of ARF6- and BZR1-target genes.

### ERF72 antagonises regulation by ARF6 and BZR1 of the transcription of *BEE3* and *XTH7*

**Fig. 4.**
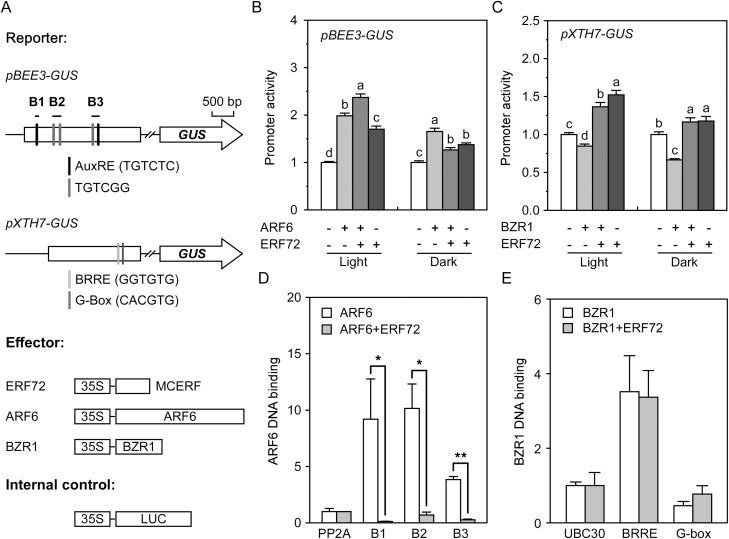
ERF72 antagonises the regulation by ARF6 and BZR1 of the transcription of *BEE3* and *XTH7*, respectively. (A) Schematic diagram of the *BEE3* promoter, *XTH7* promoter, and constructs used. LUC, firefly luciferase. GUS, beta-glucuronidase. Black and grey lines in the *BEE3* promoter region indicate the AuxRE (TGTCTC) and TGTCGG elements, respectively. Boxes B1–B3 indicate fragments that contain the AuxRE element, TGTCGG element, or both. Light grey and grey lines in the *XTH7* promoter region indicate the BRRE element (GGTGTG) and G-Box element (CACGTG), respectively. (B) Effect of ERF72 and ARF6 on transcriptional regulation of the *BEE3* promoter in Arabidopsis mesophyll protoplasts under light or dark conditions for 48 h. Promoter activity is expressed as the ratio of GUS to LUC activity. Data are means (±SD) (*n*=3). Different letters indicate significant differences at *P*<0.05 according to Tukey’s test. (C) Effect of ERF72 and BZR1 on transcriptional regulation of the *XTH7* promoter in Arabidopsis mesophyll protoplasts under light or dark conditions for 48 h. Promoter activity is expressed as the ratio of GUS to LUC activity. Data are means (±SD) (*n*=3). Different letters indicate significant differences at *P*<0.05 according to Tukey’s test. (D) Binding of the B1–B3 boxes to ARF6 was suppressed by ERF72. Relative enrichment of fragments of B1–B3 and a region of *PP2A* (At1g69960) used as a control were detected using chromatin immunoprecipitation (ChIP)-qPCR. (E) Binding of the BRRE and G-Box elements to BZR1 was not affected by ERF72. Relative enrichment of the BRRE and G-Box elements and a region of *UBC30* (At5g56150) used as a control were detected using ChIP-qPCR. Data in (D, E) are means (±SD) of three biological replicates. Significant differences were determined by one-way ANOVA: *P<0.05,; **P<0.01.

To evaluate the role of ERF72 in the transcriptional activity of ARF6 and BZR1, we selected the ARF6-target gene *BR-enhanced expression 3* (*BEE3*) and the BZR1-target gene *xyloglucan endo/transglycosidase hydrolase 7* (*XTH7*), which are related to cell elongation (Rose *et al.*, 2002; Baumann *et al.*, 2007; Cifuentes-Esquivel *et al.*, 2013). Using the *BEE3* promoter reporter system transiently transformed into Arabidopsis mesophyll protoplasts, we found that ARF6 or ERF72 alone activated the *BEE3* promoter under both light and dark conditions (Fig. 4A, B). However, when *ARF6* was co-transformed with *ERF72* activation of the reporter gene driven by the *BEE3* promoter was enhanced under light conditions but weakened under dark conditions (Fig. 4B). BZR1 is a transcriptional repressor (He *et al.*, 2005), and it inhibited *XTH7* promoter activity under both light and dark conditions (Fig. 4A, C). However, this repression was disrupted by its co-transformation with *ERF72*. Moreover, ERF72 was capable of activating the *XTH7* promoter under both light and dark conditions (Fig. 4C). In addition, the expression of *BEE3* and *XTH7* was decreased in 2-DAG seedlings of *35S::MAERF* plants compared with the wild type and *erf* under dark conditions (Supplementary Fig. S3B). These results indicated that ERF72 antagonised the transcriptional function of ARF6 and BZR1 in Arabidopsis in a light-/dark-dependent manner. Furthermore, four GCC-like boxes in the promoter regions of *BEE3* (G1 and G2) and *XTH7* (G3 and G4) were predicted to bind ERF72 (Supplementary Fig. S4A). However, the GUS activities of G1 to G4 (Supplementary Fig. S4B) were negligible (Supplementary Table S4), indicating that these GCC-like boxes were not functional. Therefore, ERF72 may regulate the transcription of *BEE3* and *XTH7* by interacting directly with ARF6 or BZR1 in Arabidopsis.

ARF6 binds to the AuxRE (TGTCTC) and TGTCGG elements in the promoter sequences of target genes, and BZR1 binds to the BRRE (GGTGTG) and G-box (CACGTG) elements (He *et al.*, 2005; Oh *et al.*, 2014). A promoter element analysis revealed that the *BEE3* promoter contains two AuxRE elements and three TGTCGG elements (B1, B2, and B3 fragments), and the *XTH7* promoter contains one BRRE element and one G-box (Fig. 4A). ChIP-qPCR showed that the B1–B3 fragments were enriched by ARF6-MYC compared with the negative control (*PP2A*), but this was markedly depressed by the co-expression of ARF6-MYC and ERF72-HA (Fig. 4D), suggesting that in the presence of ARF6, ERF72 down-regulates the expression of *BEE3*. In addition, the BRRE element-containing fragment, but not the G-box-containing fragment, of the *XTH7* promoter was enriched by BZR1-MYC, but unaffected by the co-expression of BZR1-MYC and ERF72-HA (Fig. 4E), indicating that ERF72 ameliorated the inhibition of BZR1 for expression of *XTH7* through an unknown pathway, without affecting its binding to the *XTH7* promoter.

### Light regulates the transcription and subcellular location of *ERF72*

**Fig. 5.**
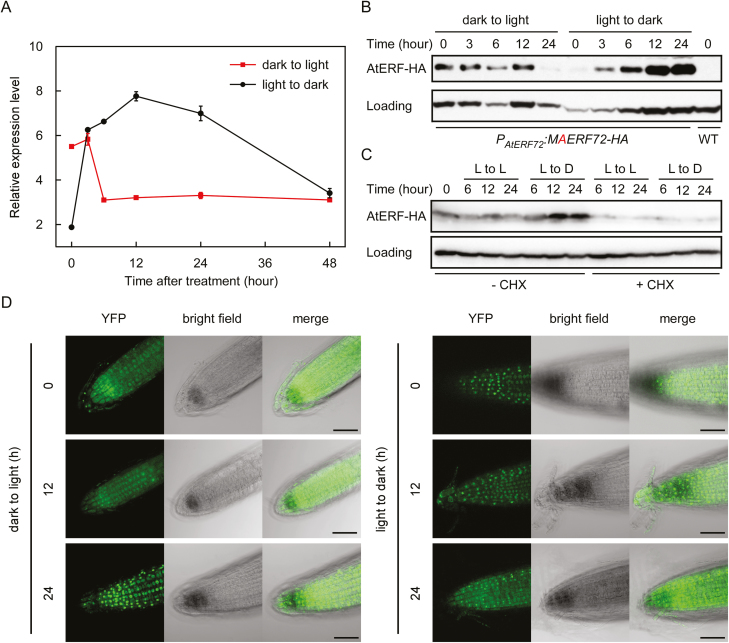
Effect of light on the transcription of *ERF72* and subcellular localisation of ERF72. (A) The expression level of *ERF72* was determined by qRT-PCR. Seedlings were grown under continuous light or dark conditions for 7 d and then transferred to the opposite conditions for the indicated times. *ERF72* expression was normalised to that of *Actin2*. Data are means (±SD) of three biological replicates. (B) Immunoblotting of ERF72 using a HA antibody in *P*_*ERF72*_*::MAERF72-HA* transgenic seedlings in the *erf* background under the same conditions as in (A). An anti-Actin antibody served as a control. WT, wild-type. (C) Immunoblotting of ERF72 using a HA antibody in *P*_*ERF72*_*::MAERF72-HA* transgenic seedlings treated with 100 µM cycloheximide (+CHX) or buffer only (–CHX). Seedlings were grown in continuous light for 7 d and transferred to half-strength MS medium with or without CHX under light or dark conditions for the indicated times. An anti-Actin antibody served as a control. (D) The subcellular localisation of ERF72 in Arabidopsis roots during the light-to-dark transition. Seedlings overexpressing *35S::MAERF-YFP* were grown under continuous light or dark conditions for 4 d and then transferred to the opposite conditions for the indicated times. Scale bars are 50 μm.

The role of overexpression of *MAERF* in photomorphogenesis in the dark led us to investigate the expression of *ERF72* in response to different light conditions. As anticipated, the expression level of *ERF72* in light-grown seedlings was lower than that in dark-grown seedlings. After seedlings were transferred to dark conditions, *ERF72* expression increased gradually for 12 h and then decreased up to 48 h (Fig. 5A). In contrast, after seedlings were transferred from dark to light conditions, the *ERF72* mRNA level decreased dramatically within 6 h and a low level was maintained up to 48 h (Fig. 5A). Consistent with our qRT-PCR results, the ERF72 protein level was reduced in *P*_*ERF72*_*::MAERF-HA* seedlings transferred from dark to light conditions and increased when the seedlings were transferred from light to dark conditions (Fig. 5B). A protein synthesis inhibitor, cycloheximide (CHX), was used to determine whether the increased ERF72 protein level was due to the increasing *ERF72* mRNA level in seedlings transferred from light to dark conditions. As shown in Fig. 5C, in the presence of CHX, the level of ERF72 protein was decreased in seedlings transferred from light to dark or between different light conditions. These results suggested that the increase in ERF72 protein was caused primarily by the increased mRNA abundance during the light-to-dark transition.

A previous study had shown that ERF72 translocates from the cytoplasm to the nucleus in response to hypoxia (Abbas *et al.*, 2015). In a similar manner, the subcellular localisation of stabilised ERF72 (MAERF-YFP) was changed from the cytoplasm to the nucleus in Arabidopsis roots during the dark-to-light transition, and this stabilised ERF72 was removed from the nucleus through a mechanism unrelated to the N-end rule pathway, as the second amino acid of ERF72, which is normally cysteine, was mutated to alanine (Fig. 5D). In summary, these results indicated that light inhibited the transcription of *ERF72* and triggered the translocation of ERF72 from the cytoplasm to the nucleus in Arabidopsis seedlings.

### *ERF72* is a target gene of EIN3

**Fig. 6.**
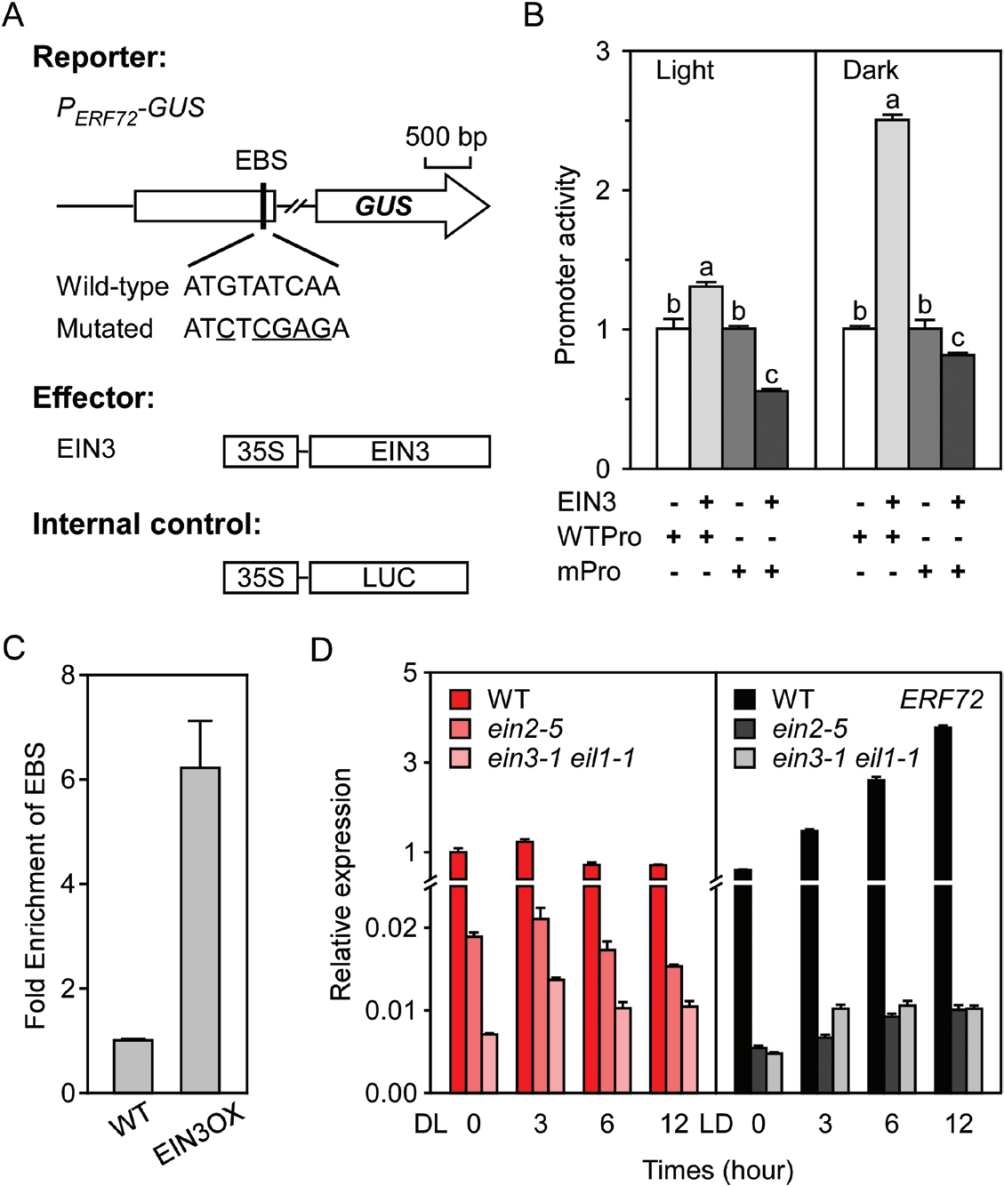
*ERF72* is a target gene of EIN3. (A) Schematic diagrams of the *ERF72* promoter (*pERF72*) and constructs used in this assay. EBS, EIN3 binding site. The mutated reporter construct harboured a mutated EBS of the *ERF72* promoter. LUC, firefly luciferase. (B) EIN3 activates the *ERF72* promoter by binding to the EBS under both light and dark conditions. Promoter activities of the wild-type (WT) or mutated *P*_*ERF72*_ are presented as the ratio of GUS to LUC activity. Data are means (±SD) (*n*=3). Different letters indicate significant differences at *P*<0.05 according to Tukey’s test. (C) EIN3 binds to the EBS site of the *ERF72* promoter. WT and *EIN3OX* seedlings were grown under dark conditions for 1 week and then subjected to chromatin immunoprecipitation (ChIP)-qPCR of EIN3 binding to the indicated promoter region. Data are means (±SD) of three biological repeats. (D) Relative expression of *ERF72* in WT, *ein2-5*, and *ein3-1 eil1-1* plants during the dark-to-light (DL) or light-to-dark (LD) transition. Seedlings were grown under continuous dark or light conditions for 7 d, then transferred to the opposite conditions for the indicated times. *ERF72* expression was normalised to that of *actin2*, and the relative expression of *ERF72* under dark conditions was set as 1.0. Data are means (±SD) of three biological replicates.

Previous research has shown that *ERF72* is a candidate target gene of EIN3 (Chang *et al.*, 2013), and its promoter region contains a putative EIN3-binding site (EBS) (Fig. 6A). *P*_*ERF72*_*::GUS* activity analysis showed that EIN3 activated the transcription of the WT *ERF72* promoter, but not the EBS-mutated promoter. Moreover, the activation activity of EIN3 was greater under dark than light conditions (Fig. 6B). ChIP-qPCR analysis showed that EIN3 binded the EBS fragment of the *ERF72* promoter (Fig. 6C). qRT-PCR analysis showed that the expression level of *ERF72* was markedly lower in *ein2-5* and *ein3-1 eil1-1* mutant seedlings than in the WT under both dark and light conditions. However, the expression trend was similar to the WT in *ein2-5* and *ein3-1 eil1-1* seedlings during the light-to-dark transition (Fig. 6D). These results demonstrated that *ERF72* was the target gene of EIN3 and that dark-induced transcription of *ERF72* was mediated by the EIN2-EIN3/EIL1 pathway in Arabidopsis.

### The expression of *BEE3* and *XTH7* is also regulated by the EIN2-EIN3/EIL1 pathway

**Fig. 7.**
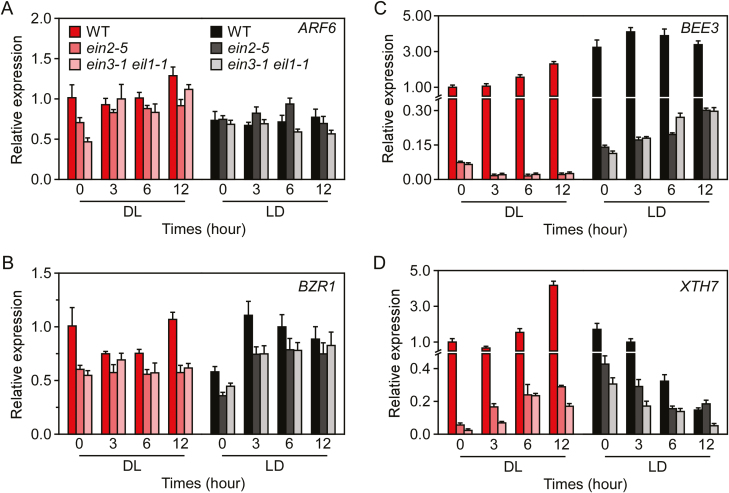
The expression of *BEE3* and *XTH7*, but not *ARF6* and *BZR1*, is regulated by the EIN2-EIN3/EIL1 pathway during the dark-to-light (DL) or light-to-dark (LD) transition. Wild-type (WT), *ein2-5*, and *ein3-1 eil1-1* seedlings were grown under continuous dark or light conditions for 7 d, then transferred to the opposite conditions for the indicated times. The relative expression levels of *ARF6, BZR1*, *BEE3*, and *XTH7* were separately normalised to that of *Actin2*. The expression level under dark conditions was set as 1.0. Data are means (±SD) of three biological replicates.

We performed qRT-PCR to investigate the role of the EIN2-EIN3/EIL1 pathway in the transcriptional regulation of *ARF6* and *BZR1*, and their target genes *BEE3* and *XTH7*. The expression level of *ARF6* was lower in *ein3-1 eil1-1* seedlings than that in the WT in dark conditions, and then it increased to the same level as the WT after transferring to light for 3 h. However, the expression of *ARF6* in *ein2-5* was not significantly different to that of the WT and *ein3-1 eil1-1* because the value change of expression level of *ARF6* in *ein2-5* was less than two-fold that in the WT and *ein3-1 eil1-1* during the dark-to-light transition. In addition, the expression of *ARF6* in *ein2-5* and *ein3-1 eil1-1* seedlings was similar to that in the WT during light-to-dark transition (Fig. 7A). At the same time, the expression of *BZR1* in *ein2-5* and *ein3-1 eil1-1* seedlings showed no significant difference with that in the WT during dark-to-light transition. However, the expression level of *BZR1* was lower in light conditions than in the dark in WT, *ein2-5*, and *ein3-1 eil1-1* seedlings, but it increased to the same level after transferring to dark conditions for 3 h (Fig. 7B). These results suggested that *ARF6* could be regulated by the ethylene signalling pathway and that *BZR1* was mainly regulated by light/dark conditions.

In addition, the expression levels of *BEE3* and *XTH7* were dramatically lower in *ein2-5* and *ein3-1 eil1-1* seedlings than in the WT under both dark and light conditions (Fig. 7C, D), which was similar to the expression patterns of *BEE3* and *XTH7* in 2-DAG seedlings of *35S::MAERF* plants under dark conditions (Supplementary Fig. S3B). We also found that the expression level of *BEE3* in the WT was increased after seedlings were transferred from dark to light conditions, and fluctuated slightly after seedlings were transferred from light to dark conditions. However, the level of *BEE3* in *ein2-5* and *ein3-1 eil1-1* seedlings was gradually decreased during dark-to-light transition and increased during light-to-dark transition (Fig. 7C). In contrast, the expression of *XTH7* was increased during dark-to-light transition and decreased during light-to-dark transition in all the genotypes (Fig. 7D). These results indicated that ethylene regulated the transcription of *BEE3* and *XTH7* in Arabidopsis via the EIN2-EIN3/EIL1 pathway, which was further balanced through the BAP/DE module according to light/dark conditions.

## Discussion

Ethylene is responsible for the triple-response phenotype of dark-grown Arabidopsis seedlings, which is characterised by exaggerated curvature of the apical hook, radial swelling of the hypocotyl, and inhibition of hypocotyl and root growth (Ecker, 1995; Benavente and Alonso, 2006). Previous studies have shown that ethylene, through EIN3/EIL1, activates two distinct pathways: the PIF3-dependent growth-promoting pathway and an ERF1-mediated growth-inhibiting pathway, to regulate hypocotyl elongation in Arabidopsis (Smalle *et al.*, 1997; Zhong *et al.*, 2012).Zhong *et al.* (2014) further showed that the soil overlaying seedlings activates ethylene production to regulate emergence via EIN3/EIL1-conducted PIF3–ERF1 molecular circuitry. Moreover, Shi *et al.* (2016*a*) showed that COP1 stabilises EIN3 levels by directly targeting EBF1 and EBF2 for ubiquitination to induce ethylene-mediated hypocotyl elongation and seedling emergence from the soil. In our study, we found that *ERF72* is another target gene of EIN3/EIL1 and the expression of *ERF72* is up-regulated during light-to-dark transition, indicating that this transition, similar to overlaying soil, induces ethylene production. We further found that ERF72 interacts with BZR1 and ARF6 to connect the ethylene-, BR-, and auxin-signalling pathways in light-regulated hypocotyl elongation of Arabidopsis seedlings. These results provide novel insights into the role of the ethylene signalling pathway in regulating seedling photomorphogenesis.

Previous studies have shown that members of the group-VII ERF (ERF-VII) family are involved in hypoxia-induced shoot elongation and photomorphogenesis in plants (Gibbs *et al.*, 2014; Giuntoli *et al.*, 2014; Sasidharan and Voesenek, 2015; Voesenek and Bailey-Serres, 2015). Under deep-water conditions, ethylene accumulation induces the expression of two ERF-VII genes, *SNORKEL1* and *SNORKEL2*, which triggers GA-mediated internode elongation in rice (Hattori *et al.*, 2009). In addition, *SUB1A-1*, which is located in the *SUBMERGENCE1* (*SUB1*) locus (another member of the ERF-VII family that also contains *SUB1B* and *SUB1C*), is reported to be a primary determinant of enhanced survival of completely submerged rice plants (Xu *et al.*, 2006). SUB1A limits shoot elongation by promoting accumulation of the GA-response transcriptional inhibitors *SLENDER RICE1* (*SLR1*) and *SLENDER RICE-LIKE1* (*SLRL1*) and concomitantly diminishing the expression of GA-inducible genes in submerged conditions in rice (Fukao and Bailey-Serres, 2008). Arabidopsis has five *ERF-VII* genes, *ERF71*–*75* (Nakano *et al.*, 2006), the products of which contain a characteristic conserved motif, the MC-dipeptide, at the amino terminus that leads to proteolysis by the N-end rule pathway (Gibbs *et al.*, 2011). The stability of ERF-VIIs is enhanced by NO signalling, and these factors are involved in regulation of seed germination, stomatal closure, and hypocotyl elongation (Gibbs *et al.*, 2014), as well as hypoxia-regulated apical hook development after germination under dark conditions in Arabidopsis. Thus, to protect the stem-cell niche, plants monitor soil oxygen content after germination by means of hypoxia-stabilised ERF-VIIs (Abbas *et al.*, 2015). However, not all ERFs that contain the MC-dipeptide are degraded by the N-end rule pathway; for example, SUB1A in rice (Gibbs *et al.*, 2011), which plays different roles in response to abiotic stresses and developmental cues (Fukao *et al.*, 2011, 2012). In our study, transgenic Arabidopsis overexpressing *MAERF*, but not those expressing *MCERF*, exhibited photomorphogenesis-related phenotypes under dark conditions (Fig. 1). These results suggest that the degradation of ERF72 through the N-end rule pathway is involved in the regulation of early seedling development in Arabidopsis.

To determine the mechanism by which ERF72 regulates hypocotyl elongation in Arabidopsis seedlings, we performed RNA-seq of dark-grown *35S::MAERF*, *erf*, and WT seedlings (Fig. 2). Light-regulated and ARF6- and BZR1-target genes were enriched in ERF72-responsive DEGs, indicating that ARF6, BZR1, and ERF72 integrate the light and hormone signals that regulate gene expression during skotomorphogenesis. Previous research has shown that ARF6, BZR1, and PIF4 interact with each other to promote hypocotyl elongation by co-activating numerous shared target genes with known functions in cell elongation (Oh *et al.*, 2014). Furthermore, DELLAs, as transcriptional inhibitors, form complexes with ARF6, BZR1, and PIF4 to create the BZR-ARF-PIF/DELLA (BAP/D) transcriptional module that regulates the expression of cell elongation-related genes, which in turn mediates the co-operative regulation of Arabidopsis morphogenesis by BR, auxin, GA, and light signals (Bai *et al.*, 2012; Oh *et al.*, 2014; Chaiwanon *et al.*, 2016). In addition, ethylene stimulates cell wall acidification and induces production of cell wall modification proteins to facilitate rapid elongation of submerged *Rumex palustris* petioles (Voesenek and Bailey-Serres, 2015). Therefore, we evaluated the promoter activity of the ERF72-responsive DEGs, *BEE3* and *XTH7*, which are target genes of ARF6 and BZR1, respectively. The results showed that ERF72 interacts with ARF6 and BZR1 (Fig. 3), inhibiting the transcriptional activity of ARF6 by suppressing its binding to the *BEE3* promoter and directly antagonising the effect of BZR1 on transcription of *XTH7*. In addition, a previous study showed that DELLAs interact with and inhibit DNA binding by ERF72 (Marín-de la Rosa *et al.*, 2014). Therefore, we propose that the revised BAP/DE (BZR-ARF-PIF/DELLA-ERF) module integrates ethylene, auxin, BR, GA, and light signals to regulate hypocotyl elongation during seedling photomorphogenesis, suggesting that crosstalk among these four proteins affects downstream gene expression related to hypocotyl cell elongation.

**Fig. 8.**
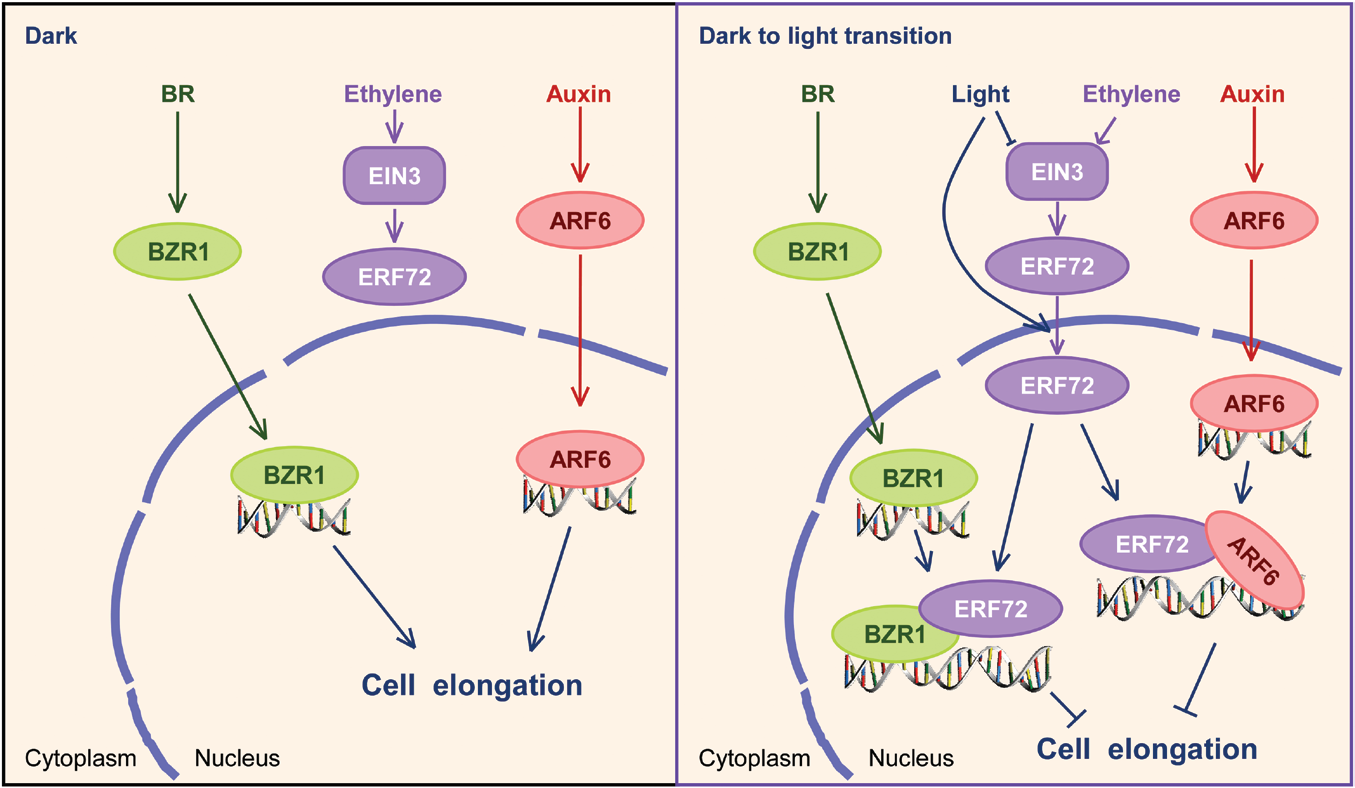
Crosstalk among the auxin, brassinosteroid (BR), and ethylene signalling pathways, mediated by ARF6, BZR1, and ERF72, regulates hypocotyl growth and photomorphogenesis in Arabidopsis seedlings.

*ERF72* is a candidate target of EIN3 (Chang *et al.*, 2013). Our results showed that EIN3 binds the EBS fragment of the *ERF72* promoter to activate the transcription of *ERF72* under dark conditions (Fig. 6); indeed, the expression level of *ERF72* was markedly lower in *ein2-5* mutant and *ein3-1 eil1-1* double-mutant seedlings than in the WT under both dark and light conditions. These results demonstrated that *ERF72* is a target gene of EIN3, and the transcription of *ERF72* is regulated by the EIN2-EIN3/EIL1 pathway in Arabidopsis. Previous studies have shown that COP1 stabilises EIN3 levels by directly targeting EBF1 and EBF2 for ubiquitination under dark conditions (Shi *et al.*, 2016*a*). However, phyB interacts with EIN3 and EBF1/EBF2 in a light-dependent manner and stimulates degradation of EIN3 by SCF^EBF1/EBF2^ E3 ligases to attenuate ethylene-mediated responses (Shi *et al.*, 2016*b*). In our study, dark conditions induced transcription of *ERF72* and increased the abundance of ERF72 in Arabidopsis seedlings (Fig. 5). These results are in agreement with the stability of EIN3 under different light conditions. In addition, Li and Chye (2004) reported that AtEBP/ERF72 is transported to the plasma membrane by interacting with acyl-CoA-binding protein 2 (ACBP2) after transient co-expression in tobacco leaves. Here, we showed that ERF72-YFP fluorescence was dispersed in the cytoplasm under dark conditions and aggregated in the nucleus under light conditions; this nuclear localisation facilitates the function of ERF72. Therefore, we propose a mechanism by which the light, auxin, BR, and ethylene signalling pathways interplay to regulate hypocotyl growth and seedling morphogenesis (Fig. 8). In dark-grown seedlings, EIN3 mediates ethylene-induced expression of *ERF72*, despite ERF72 being localised in the cytoplasm, while ARF6 and BZR1 in the nucleus regulate the expression of target genes. This results in expression of a large number of cell elongation-related genes to mediate skotomorphogenic growth. Following exposure of dark-grown seedlings to light, ERF72 is translocated to the nucleus by an unknown mechanism, and interacts with ARF6 and BZR1 to attenuate the transcriptional regulation of target genes of ARF6 and BZR1. As a result, hypocotyl growth is inhibited and seedlings undergo photomorphogenesis. In our study, overexpression of *MAERF* led to accumulation of ERF72 in nuclei in dark-grown transgenic seedlings, which limited regulation by ARF6 and BZR1 of the expression of cell elongation-related genes, resulting in photomorphogenic growth. Our results suggest that light, auxin, BR, and ethylene signalling through the revised BAP/DE module play harmonious roles in the regulation of hypocotyl elongation in Arabidopsis seedlings.

Interestingly, the expression of *BEE3* and *XTH7* was dramatically down-regulated in *ein2-5* and *ein3-1 eil1-1* seedlings compared to the WT under both dark and light conditions. Several putative EBS-like sites were predicted in the region upstream of the ATG site in the *BEE3* and *XTH7* promoters, which require further confirmation by a GUS activity assay (Supplementary Fig. S5). These results indicated that *BEE3* and *XTH7* are the potential target genes of EIN3. However, the expression of *BEE3* and *XTH7* were also regulated by different light conditions in *ein2-5* and *ein3-1 eil1-1* seedlings. In addition, we found that *BEE3* and *XTH7* are the target genes of ARF6 and BZR1, respectively. And ERF72, as one of the target genes of EIN3, interacts with ARF6 and BZR1 to separately regulate the transcriptional activity of ARF6 on *BEE3* and that of BZR1 on *XTH7*. We also found that the expression of *BEE3* and *XTH7* was dramatically decreased in 2-DAG seedlings of *35S::MAERF* plants compared with the WT and *erf* under dark conditions. These results suggested that the expression of *BEE3* and *XTH7* are fine-tuned by light and phytohormones via merging the EIN2-EIN3/EIL1 pathway and BAP/DE module during seedling photomorphogenesis in Arabidopsis.

## Supplementary data

Supplementary data are available at *JXB* online.

Table S1. List of primers used in this study.

Table S2. RNA-seq data and its alignment to the Arabidopsis reference genome.

Table S3. List of differentially expressed genes.

Table S4. Raw data for promoter activity analysis.

Fig. S1. *ERF72* expression in the WT, *erf* mutant, *35S::MAERF*, and *35S::MCERF* transgenic lines.

Fig. S2. Cotyledon development of WT, *erf*, and transgenic plants under dark conditions.

Fig. S3. Expression of *ARF6*, *BZR1*, *BEE3*, and *XTH7* detected in seedlings of WT, *erf*, and *35S::MAERF* grown under dark conditions.

Fig. S4. Locations of GCC-like boxes in the *BEE3* and *XTH7* promoters and the constructs used for GUS assays.

Fig. S5. Locations of the putative EBS-like sites in the *BEE3* and *XTH7* promoters.

Supplementary Material

Supplementary Table S3

## Abbreviations:

ERF72: ethylene response factor 72
ARF6: auxin response factor 6
BZR1: brassinazole-resistant 1
BAP/DE: BZR-ARF-PIF/DELLA-ERF
BR: brassinosteroid
GA: gibberellin.
