# Supplementary material for: ERF72 interacts with ARF6 and BZR1 to regulate hypocotyl elongation in Arabidopsis
Source: J Exp Bot. 2018 Jun 12;69(16):3933–47. doi: 10.1093/jxb/ery220 (PMC6054149; doi:10.1093/jxb/ery220)

Supplementary Information

Supplemental Table S1. List of primers used in this study.

Supplemental Table S2. Our RNA-seq data and their alignment to the *Arabidopsis* reference genome.

Supplemental Table S3. List of differentially expressed genes (DEGs). Genes with different coloured backgrounds represent DEGs in the clusters in Figure 2B: Pink background, DEGs in cluster a. Sky blue, DEGs in cluster b. Mint green, DEGs in cluster c. Cyan, DEGs in cluster d. Purple, DEGs in cluster e. Light brown, DEGs in cluster f. Light grey, DEGs in cluster g. Orange, DEGs in cluster h. FPKM, fragments per kilobase of transcript sequence per million base pairs.

Supplemental Table S4. The raw data of promoter activity analysis using the constructs shown in Figure 4B, 4C, and S4. The GUS-0 and GUS-60 columns refer to the initial and 60-min fluorescence values, respectively, determined by GUS assay, and the LUC column shows the fluorescence values obtained by LUC assay.

Supplemental Figure S1. *ERF72* expression in the WT, *erf* mutant, *35S:MAERF*, and *35S:MCERF* transgenic lines. (A) Diagram of the T-DNA insertion mutant *erf* (Stock no. CS849696). Black boxes represent exons, and lines between boxes indicate introns. The triangle indicates the position of the T-DNA insertion in *erf*. ATG, start codon; TAA, stop codon. (B) The *ERF72* mRNA level was detected via RT-PCR in 7-day-old seedlings of the WT, *erf*, *35S:MAERF*, and *35S:MCERF* transgenic lines. *Actin 2* was used as a control. (C) The ERF72 protein level was detected via western blotting in WT, *erf*, *35S:MAERF*, and *35S:MCERF* transgenic lines. Seven-day-old seedlings grown in darkness were incubated with or without 50 μM MG132 for 24 h. Lines #9 and #11 are independent lines overexpressing stable *ERF72* in

a WT background (*35S:MAERF*); #95 and #107 are independent lines overexpressing WT *ERF72* in a WT background (*35S:MCERF*). Actin was used as the loading control. (D) Western blot of the same sample as (C), which was colour-developed and exposed for a long time to visualise protein bands.

Supplemental Figure S2. Cotyledon development of WT, *erf*, and transgenic plants under dark conditions. #9 and #11, independent lines overexpressing stable *ERF72* driven by the 35S promoter in a WT background (*35S:MAERF*); #95 and #107, independent lines overexpressing the WT *ERF72* in a WT background (*35S:MCERF*) under the same conditions. DAG, days after germination. Bar, 1 mm.

Supplemental Figure S3. Expression of *ARF6*, *BZR1*, *BEE3*, and *XTH7* was detected in 2-DAG seedlings of WT, *erf,* and *35S:MAERF* grown under dark conditions by qRT-PCR. The expression levels were normalised to that of *Actin2*. Data are means ± SD of three biological replicates.

Supplemental Figure S4. Locations of GCC-like boxes in the *BEE3* and *XTH7* promoters and the constructs used for GUS assays. (A) Locations of GCC-like boxes in the region upstream of the ATG site in the promoters of *BEE3* and *XTH7*. Light and dark grey boxes, sequences of putative GCC-like boxes on the plus and minus strands, respectively, and the distance to the ATG codon. G1 to G4, fragments used for plasmid construction. (B) Constructs used for GUS assays. LUC, firefly luciferase; GUS, beta-glucuronidase.

Supplemental Figure S5. Locations of the putative EBS-like sites in the *BEE3* and *XTH7* promoters. Light and dark grey boxes indicate the sequences of EBS-like sites on the plus and minus strands, respectively, and the distance to the ATG codon of *BEE3* and *XTH7*.

Table S1 List of primers used in this study.

Gene	Oligo name	Sequence (5’-3’)	Purpose
AtERF72	ERF-F	CGGGATCCATGTGTGGCGGTGCTATTAT	Cloning of *AtERF72*
	ERF-R	ATAAGAATGCGGCCGCCTCATACGACGCAATGACATC	
	MAERF-F	CGGGATCCATGGCTGGCGGTGCTATTATT	Mutation of *AtERF72*
	MAERF-R	ATAAGAATGCGGCCGCCTCATACGACGCAATGACATC	
	ERF-Q-F	CCGGTGGAGTGTATAGGATTTG	qRT-PCR
	ERF-Q-R	CCAAGCTCGATATCTGCTGTT	
	ERF-P-F	CCC aagctt TCTTGGGCTGTGATTCTTAT	Cloning of the promoter of *AtERF72*
	ERF-P-R	GG ggtacc TTTCTCTGTTTGTTGCTGTGGGT	
	ERF-mP-F	TAACCGTCATCTCGAGAGGAACATA	Mutation of the promoter of *AtERF72*
	ERF-mP-R	TATGTTCCTCTCGAGATGACGGTTA	
	ERF-EBS-F	TTATGTGGATTAGCCTTCAATTTG	ChIP-qPCR for EBS in *AtERF72* promoter
	ERF-EBS-R	GCCTTTATAGGCGCAAAATAAAT	
ARF6	ARF6-F	CGGAATTCGGATCCATGAGATTATCTTCAGCTGGGTT	Cloning of *ARF6*
	ARF6-R	ATAAGAATGCGGCCGCGTAGTTGAATGAACCCCCAACT	
	ARF6-C-F	CGGAATTCGGATCCCCTCTCAGGCTTAAACGGC	Cloning for yeast two-hybrid assay
	ARF6-N-R	ATAAGAATGCGGCCGCAAAAGGAGATGGATACATAGGGA	
	ARF6-nLUC-F	CGGAATTCGGATCCATGAGATTATCTTCAGCTGGGTT	Cloning for LCI assay
	ARF6-nLUC-R	CCGCTCGAGGTAGTTGAATGAACCCCCAACT	
	ARF6-Q-F	CCGCATAGCCATCTTCTCTTT	qRT-PCR
	ARF6-Q-R	GTGGAGTCACCACCTTCAATAC	
BZR1	BZR1-F	CGGAATTCGGATCCATGACTTCGGATGGAGCTACGT	Cloning of *BZR1*
	BZR1-R	ATAAGAATGCGGCCGCACCACGAGCCTTCCCA	

	BZR1-nLUC-F	CGGAATTCGGATCCATGACTTCGGATGGAGCTACGT	Cloning for LCI assay
	BZR1-nLUC-R	TGCTCTAGAACCACGAGCCTTCCCA	
	BZR1-Q-F	TGGTGAGATAGCTGGGACTT	qRT-PCR
	BZR1-Q-R	GGTAAGATGGGATGGGACTTTG	
	BZR1-C-F	CGGAATTCGGATCCGTAACTCCATATTCATCACAGAACCA	Cloning for yeast two-hybrid assay
	BZR1-N-R	ATAAGAATGCGGCCGCTCGAGATGAAGTCCCAGCTATCT	
EIN3	EIN3-cDNA-F	AGTCGCTTTCTAGCCACGTG	Cloning of *EIN3*
	EIN3-cDNA-R	TGTTCGTTCATCCCCAAGTC	
	EIN3-F	CGGGATCCATGATGTTTAATGAGATGGGAAT	
	EIN3-R	ATAAGAATGCGGCCGCGAACCATATGGATACATCTTGCT	
BEE3	BEE3-P-F	CGGGATCCCCAAGGTCGTAGAGGAGGCG	Cloning the promoter of *BEE3* and its GCC-like boxes
	BEE3-P-R	TTGGCGCGCCAAATTGGTGAAAATAGAAAGTGGCT	
	BEE3-PG1-F	AGGCGCGCCACTAGTTCCAAGGTCGTAGAGGAGGC	
	BEE3-PGI-R	GCTCTAGAAAGGAAGACATCGAAATGGAGTTC	
	BEE3-PG2-F	AGGCGCGCCACTAGTAGAAGTTTGCTTAAACAGTGCTC	
	BEE3-PG2-R	GCTCTAGATTGTATAGGAGTGGGTCACACAT	
	BEE3-PB1-F	AAGATCAAAACATGTAAGGTCACCC	ChIP-qPCR for ARF6 binding sites in *BEE3* promoter
	BEE3-PB1-R	GATCTCAAATATAACGAACGATCCA	
	BEE3-PB2-F	TGACCATAACAAAAGTTGAACAAGC	
	BEE3-PB2-R	GGTTTTAGGCATGTACCTGACTTGT	
	BEE3-PB3-F	CAAGTTAGATTGAGTCTTGAATGCA	
	BEE3-PB3-R	TAAGTTCGTTTTAACTTGGTTTGGT	
XTH7	XTH7-P-F	CCCAAGCTTGCACATACAAAACTGGCTAAAC	Cloning the promoter of *XTH7* and its GCC-like boxes
	XTH7-P-R	AGGCGCGCCTGTTGCTTATGATGATGAGTTTG	
	XTH7-PG3-F	CCCAAGCTTCTCGAGTTTACCAAATCTACTATTAACGAA	

	XTH7-PG3-R	ACGCGTCGACGTCTTTGAATGATTTTAACTTTTG	
	XTH7-PG4-F	CCCAAGCTTCTCGAGTGTGAGTTTAACCTTGCCAT	
	XTH7-PG4-R	ACGCGTCGACAGCCAATTGTATACTTCAACTTT	
	XTH7-PBRRE-F	CAAACGAATATAAACATGTATTTAAGG	ChIP-qPCR for BZR1 binding sites in *XTH7* promoter
	XTH7-PBRRE-R	CGATCGTCGTATCTTTCGTAGAT	
	XTH7-PGBOX-F	TGCGAGACATAGCAGAGTAATG	
	XTH7-PGBOX-R	GAAGCATAAGAATTTCCCAGTGTATAG	
PP2A	PP2A-Q-F	CGGCTTTCATGATTCCCTCT	ChIP-qPCR
	PP2A-Q-R	GCCTTAAGCTCCGTTTCCTACTT	
CNX5	CNX5-Q-F	TGACATCGTCTTCTTTGCTGCTTCT	
	CNX5-Q-R	TGCCTTCTTGAGCTTTAACTCTTCC	
UBC30	UBC30-NC-F	GTCTCTGGTGATTCTGAGATGG	
	UBC30-NC-R	AGACATGGGACGTTTGTACTT	
Actin2	ACT-Q-F	TAACAGGGAGAAGATGACTCAGATCA	qRT-PCR
	ACT-Q-R	AAGATCAAGACGAAGGATAGCATGAG	

Table S2. Our RNA-seq data and their alignment to the *Arabidopsis* reference genome.

Sample name	Raw reads	Clean reads	Total mapped	Multiple mapped	Uniquely mapped
WT	21792884	21335054	19198311	233717	18964594
erf	24621844	24102084	21646118	226512	21419606
35S:MAERF-9#	32551706	31974330	28271659	167911	28103748
35S:MAERF-11#	22012880	21538600	18580905	99351	18481554

Raw reads: total number of sequenced Reads.

Clean reads: total number of reads obtained from raw reads after removing adapters and low quality reads.

Total mapped: total number of clean reads that could be located to genome of Arabidopsis.

Multiple mapped: number of clean reads that could be located to more than one site of Arabidopsis genome.

Uniquely mapped: number of clean reads that could match to only one site in Arabidopsis genome.

Table S4. The raw data of promoter activity analysis using the constructs shown in Figure 4B, 4C, and S4. The GUS-0 and GUS-60 columns refer to the initial and 60-min fluorescence values, respectively, determined by GUS assay, and the LUC column shows the fluorescence values obtained by LUC assay.

**Reporter**	**Effector**			**Fluorescence value**					
	AtERF72	ARF6	BZR1	Light			Dark		
				GUS-0	GUS-60	LUC	GUS-0	GUS-60	LUC
pBEE3	-	-	-	1069.7±11.2	16510.0±131.0	362998.0±5324.3	1037.3±18.0	12986.0±86.9	331584.7±9936.4
	-	+	-	1207.7±20.5	8348.3±36.3	84625.7±2245.3	1298.3±23.6	16240.7±132.5	250614.3±8434.3
	+	+	-	1002.3±17.6	10562.3±90.6	94831.3±268.0	1116.7±18.6	10737.7±108.5	211108.0±6353.8
	+	-	-	1029.3±11.7	6017.7±54.0	69005.0±2134.2	1274.0±22.0	10896.0±91.5	194035.3±3925.0
pXTH7	-	-	-	1390.0±59.9	22129.3±115.5	261327.0±4407.2	1101.7±13.6	21253.0±210.6	313679.0±7822.6
	-	-	+	930.3±16.6	11119.3±82.1	151584.7±3884.3	1128.0±21.5	16431.3±124.7	357647.3±6308.5
	+	-	+	1091.7±16.6	13329.3±123.0	112949.3±3567.7	1135.0±20.5	15520.7±155.9	192305.3±7241.5
	+	-	-	1239.3±23.0	26234.0±260.4	206923.0±5860.0	1170.3±23.0	27982.0±243.2	354554.0±14666.6
G1	-	-	-	1267.7±24.2	1762.3± 24.0	765933.0±23473.1	1184.0±22.1	1408.3± 31.6	296097.0± 8567.1
	+	-	-	940.7±11.6	1559.0± 21.4	565142.7±21785.6	1173.7±24.0	1511.0± 28.5	473227.3±11801.8
	+	+	-	1054.7±19.0	1295.7± 21.1	207098.7± 8302.8	1150.0±27.1	1568.3± 39.3	272950.7± 7692.7
	-	+	-	1135.0±13.5	1290.3± 12.5	313331.7±10018.8	1132.3±22.6	1239.3± 24.5	125377.3± 4079.5
G2	-	-	-	1158.7±18.6	1580.7± 34.1	409603.3±16146.0	1140.3±23.0	2141.0± 47.1	218635.3± 7098.1
	+	-	-	984.7±16.6	1848.0± 36.2	804854.7±28288.7	1061.3±21.5	2045.0±106.8	467729.7±13123.6
	+	+	-	978.0±17.0	1708.0± 29.5	432279.7±18433.0	1104.3±24.0	1553.7± 36.6	198333.7± 6328.7
	-	+	-	1085.7±22.5	1555.3± 34.6	184821.7± 9650.1	1123.0±19.5	1232.0± 32.4	65633.3± 1794.1
G3	-	-	-	1144.7±17.0	7949.3± 86.7	623682.3±19929.5	1242.3±28.4	6176.0± 74.5	273618.0± 6878.8
	+	-	-	1174.3±23.5	12086.0±288.1	1014059.7±41357.2	1148.0±13.0	7920.0± 94.1	354575.7±12335.3

	+	-	+	1267.7±24.5	10148.3±104.5	624629.0±25881.4	1036.0±24.3	6379.3± 70.3	286667.0±10608.2
	-	-	+	1036.3±22.5	5912.7± 61.1	242056.7± 9594.7	993.0±25.7	2994.3± 62.9	76210.3± 2837.5
G4	-	-	-	1143.3±25.0	1748.7± 42.8	607155.3±27925.8	1056.3±22.6	1388.7± 34.1	342675.3± 9003.6
	+	-	-	1136.7±25.0	1576.3± 35.1	763777.7±38780.4	1166.7±21.5	1471.3± 30.0	446542.0±19778.3
	+	-	+	1126.7±28.9	1728.7± 40.0	690362.7±29997.2	967.7±23.1	1333.7± 30.0	361844.3±13936.2
	-	-	+	1203.0±19.1	1641.0± 33.6	417327.7±16145.5	1153.0±28.6	1282.7± 24.5	140002.7± 4931.4

Supplemental Figure S1

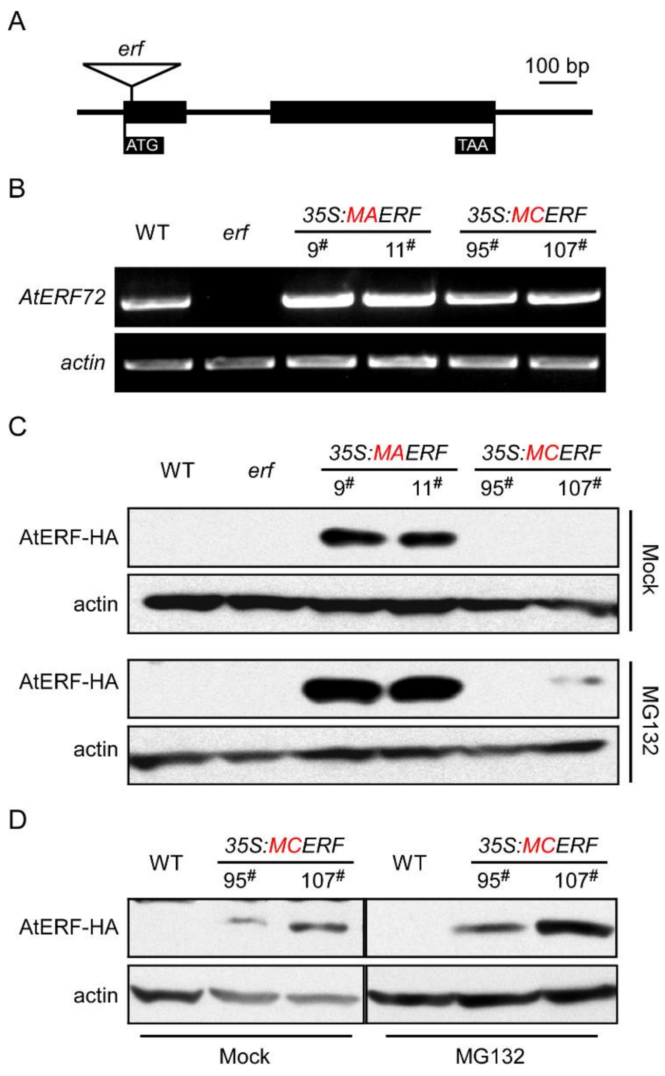

Supplemental Figure S2

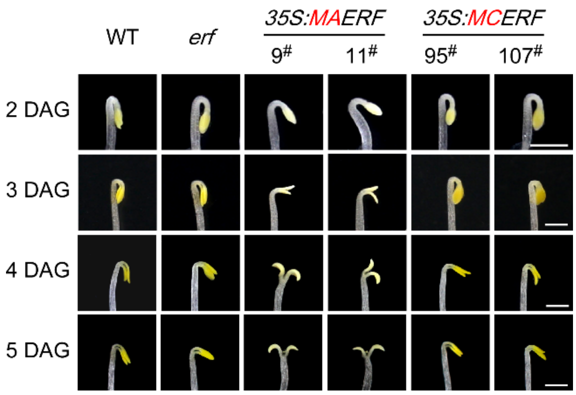

Supplemental Figure S3

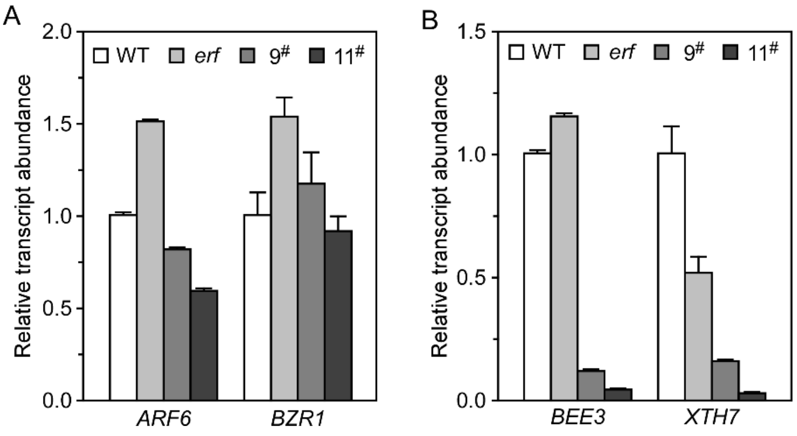

Supplemental Figure S4

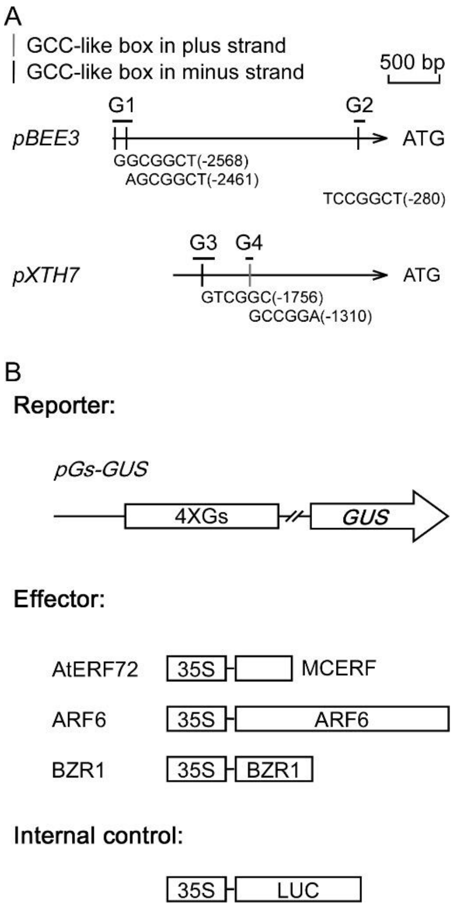

Supplemental Figure S5

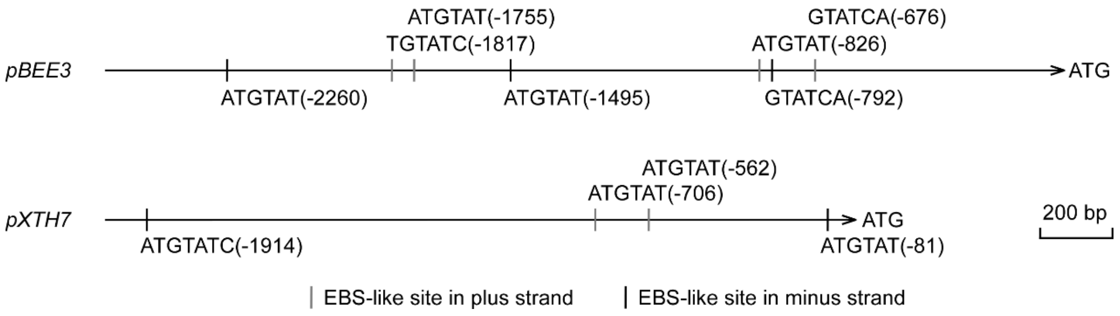

Supplement: Supplementary Material [file ery220_suppl_supplementary_material.pdf]
